# Cardiac rehabilitation and health‐related quality of life in preserved ejection fraction heart failure: A meta‐analysis

**DOI:** 10.1002/ehf2.15404

**Published:** 2025-09-03

**Authors:** Chenyao Ding, Yawen Gao, Rod S. Taylor

**Affiliations:** ^1^ School of Health and Well Being, College of Medical, Veterinary and Life Sciences University of Glasgow Glasgow UK; ^2^ Robertson Centre for Biostatistics and Primary Care and General Practice, School of Health and Well Being, College of Medical, Veterinary and Life Sciences University of Glasgow Glasgow UK

**Keywords:** cardiac rehabilitation, exercise training, health‐related quality of life, heart failure with preserved ejection fraction

## Abstract

**Aims:**

The study aims to evaluate the effects of exercise‐based cardiac rehabilitation (ExCR) on the health‐related quality of life (HRQoL) in people with heart failure preserved ejection fraction (HFpEF).

**Methods:**

This study is a systematic review and meta‐analysis. Six bibliographic databases (Medline, Embase, Web of Science, Cumulative Index of Nursing and Allied Health Literature, Cochrane CENTRAL and China National Knowledge Infrastructure database) were searched to April 2024 for randomized controlled trials (RCTs), involving adults with HFpEF undertaking ExCR compared with no exercise control. Subgroup and sensitivity analyses were conducted to explore potential sources of statistical heterogeneity.

**Results:**

Twelve RCTs recruiting a total of 1005 HFpEF patients with a median of 16 weeks follow‐up were included. Four trials defined HFpEF as an ejection fraction of ≥45% and eight trials as ≥50%. Compared with control, ExCR participation was associated with improvements in disease‐specific HRQoL as assessed by the Minnesota Living with Heart Failure Questionnaire (MLHFQ) [weighted mean difference (WMD): −6.72, 95% confidence interval (Cl): −12.00 to −1.44, *P* = 0.013] and Kansas City Cardiomyopathy Questionnaire (KCCQ) total scores (WMD: 5.34, 95% CI: 1.75 to 8.93, *P* < 0.0001) and generic HRQoL assessed by Short‐Form 36 and EQ‐5D. There was evidence (*P* ≤ 0.05) of greater improvements in MLHFQ total score with ExCR in trials with shorter exercise duration (<60 min/session), the presence of risk of bias, and larger sample size (>45 patients). Included trials were small and demonstrated substantial clinical and statistical heterogeneity with a range of: (1) population definitions (e.g., definition of HFpEF of ≥45% vs. ≥50%, level and nature of comorbidities), (2) ExCR interventions (e.g., exercise only vs. comprehensive CR programmes, different modes and intensity of exercise, centre‐ and home‐based delivery) and (3) methods of HRQoL assessment (e.g., disease specific vs. generic measure).

**Conclusions:**

This meta‐analysis of RCT evidence shows that participation in ExCR provides important gains in HRQoL of people with HFpEF. However, the results should be interpreted with caution given the substantial clinical and statistical heterogeneity. Well reported, fully powered RCTs with longer follow‐up are needed to confirm these findings.

## Graphical abstract

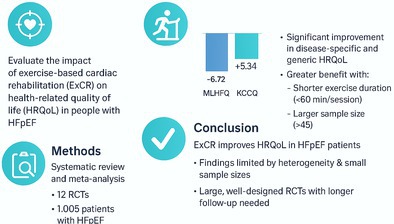

## Introduction

Heart failure (HF) affects more than 64 million people worldwide.^1^ The European Society of Cardiology (ESC) 2023 guidelines define HF with preserved ejection fraction (HFpEF) as a left ventricular ejection fraction (LVEF) of ≥50%.^2^ Currently, HFpEF accounts for approximately 50% of all incident HF, a proportion that is expected to increase with population ageing.^3^ People with HFpEF experience high rates of hospitalization, morbidity and mortality.^4^ Poorer levels of health‐related quality of life (HRQoL) in HFpEF have been reported compared with HF with reduced ejection fraction (HFrEF).^5^

A number of studies of HF therapies that have been shown to benefit people with HFrEF have had limited efficacy in HFpEF.^6^ However, there is an increasing body of evidence supporting the beneficial impact of exercise‐based cardiac rehabilitation (ExCR).^7^ ExCR involves a structured exercise programme tailored to improve patient cardiovascular health and well‐being that can delivered in inpatient, outpatient, community or home‐based setting.^8^, ^9^ It is recommended that ExCR include psychosocial or educational interventions—‘comprehensive CR’. While an individual‐participant meta‐analysis of randomized controlled trials (RCTs)^10^ shows the beneficial effects of ExCR participation on HRQoL in HFrEF, the evidence base in HFpEF remains less certain.^11^

This study aims to provide a contemporary and comprehensive update on the impact of ExCR on HRQoL in people with HFpEF by undertaking a systematic review and meta‐analysis of RCTs.

## Methods

This study was conducted and reported in accordance with the Preferred Reporting Items for Systematic Reviews and Meta‐Analyses (PRISMA) guidance.^12^

### Search strategy

The following bibliographic databases were searched: Medline, Embase, Web of Science, Cumulated Index in Nursing and Allied Health Literature (CINAHL), Cochrane Central Register of Controlled Trials (CENTRAL) and China National Knowledge Infrastructure (CNKI) over the period January 2001 to April 2024. Older studies (i.e., published before 2001) were not considered as they may not reflect contemporary clinical practice. The reference lists of included studies and relevant reviews were searched manually for potentially additional studies. Inclusion was limited to English and Chinese language studies.

### Eligibility criteria

RCTs were included if the met the following criteria: (1) adults (≥18 years) with an LVEF of ≥45% (this threshold was chosen to reflect the range of HFpEF definitions employed by previous trials); (2) participating in an ExCR programme; (3) with no exercise control group and (4) reporting HRQoL assessed using disease‐specific or generic measure at ≥12 weeks post‐randomization. Trials not available as full‐text publications were excluded. A single reviewer (C. D.) assessed trial inclusion based on review of title and abstracts or publication full text. Inclusion was checked by a second reviewer (Y. G.). Where inclusion decisions could not be resolved, a third reviewer (R. S. T.) was involved.

### Data extraction

Data extraction included study characteristics (i.e., year and county of publication, ExCR delivery model, trial follow‐up period and sample size), patient characteristics [i.e., LVEF, New York Heart Association (NYHA) class, age, gender and comorbidities], ExCR intervention details (i.e., exercise training duration, frequency, intensity and delivery setting), and HRQoL outcome measures used and their results. Where reported, we extracted both the total and subscore/domain scores of HRQoL measures.^13^, ^14^, ^15^, ^16^ Inclusion was undertaken by a single reviewer (C. D.) and checked by a second reviewer (Y. G.).

### Bias assessment

Risk of bias for each trial was assessed using the revised Cochrane tool for RCTs (RoB 2).^17^ This included the following risk of bias domains: (1) randomization process (concealment and random sequence generation); (2) deviations from intended intervention delivery; (3) outcome measurement; (4) outcome reporting. Each domain was classified as ‘low risk’ (low probability of bias affecting the outcome); ‘some concerns’ (potential bias may influence result confidence); and ‘high risk’ (high probability of bias significantly affecting confidence in results). Funnel plots and the Egger test^18^ were used to assess publication bias when there were 10 or more trials.

### Statistical analysis

Continuous variables and HRQoL effect sizes were expressed as weighted mean difference (WMD) and 95% confidence Intervals (95% CIs). Given the potential level of clinical heterogeneity across included trials due to variations in population characteristics and the nature of ExCR interventions, a random effects meta‐analysis was used. Three‐arm study results were assessed using the Cochrane ‘combining groups method’.^19^

A *Z*‐test was used to determine if the pooled WMD between ExCR and control was statistically significant at a *P* value ≤ 0.05. Cochran's *Q* test (*χ* ^2^ test) and *I* ^2^ statistic were used to quantify the level of statistical heterogeneity across included trials.^20^ An *I* ^2^ value of 25%, 50% and 75% indicated ‘low’, ‘moderate’ and ‘high’ statistical heterogeneity, respectively.

Statistical heterogeneity was explored using subgroup and sensitivity where there were 10 or more studies reporting the same HRQoL outcome.^20^ The following trial level subgroups were pre‐defined: (1) sample size (<median vs. ≥median); (2) LVEF (≥45% vs. ≥50%); (3) exercise training duration (<60 min/session vs. ≥ 60 min/session); and (4) ExCR delivery model [centre‐based, home‐based, vs. hybrid (combination of centre and home)]. Sensitivity analyses were conducted, separately pooling ‘low‐risk’ trials and trials that were judged to be either ‘some concerns or high risk’ groups.^17^ All statistical analyses were conducted using StataMP 18.

## Results

### Study selection

**Figure 1:**
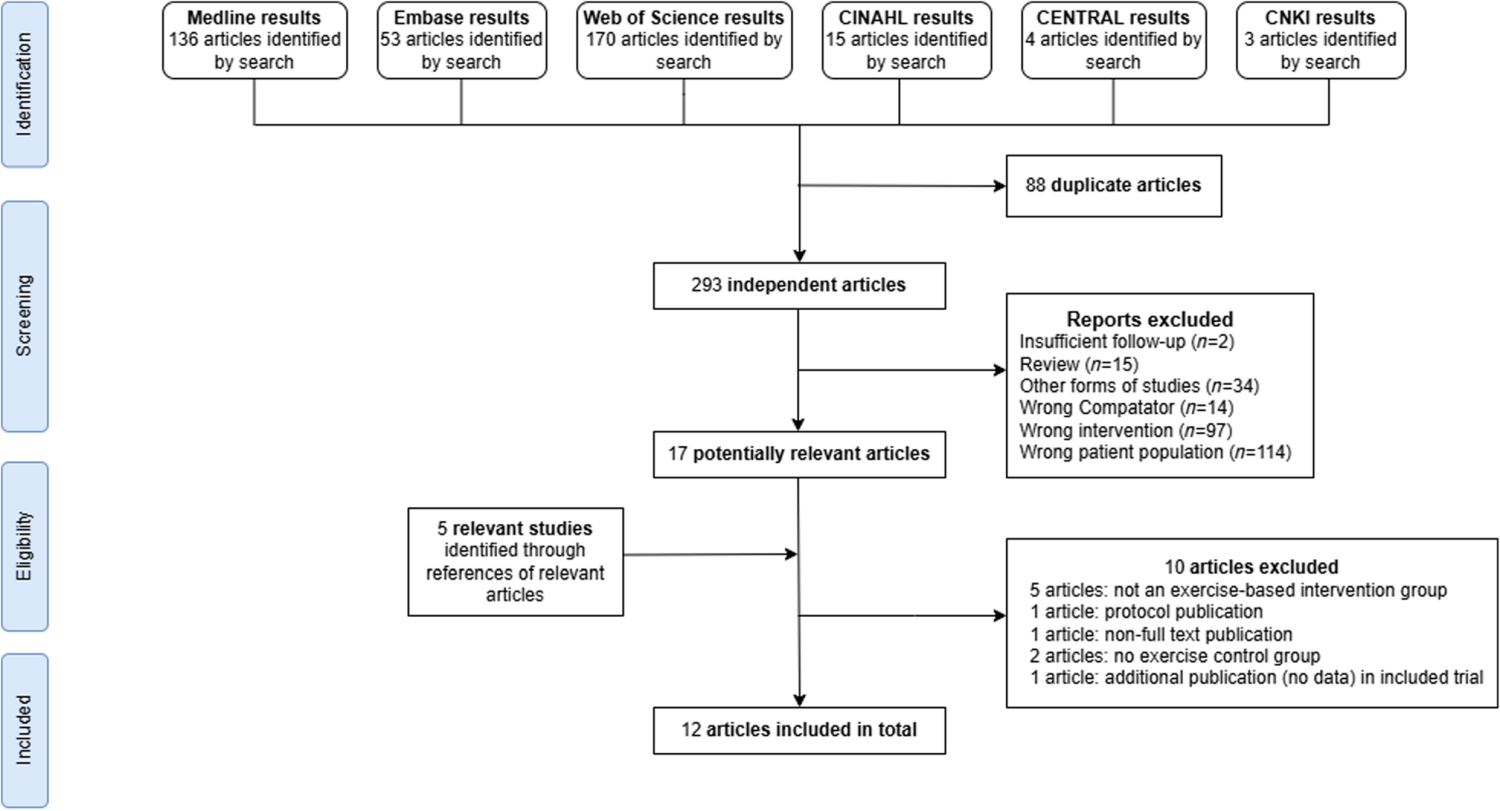
PRISMA flow diagram of study selection process.

Following the de‐duplication of the database searches, a total of 293 titles and abstracts were assessed for screening, of which 276 were excluded. Twenty‐two full‐text papers reviewed for inclusion, and 10 were excluded (see Appendix S1). Two trials^21^, ^22^ were excluded as they focused on training specifically targeting the inspiratory muscles. The remaining 13 publications included data on 12 RCTs (see *Figure* 1). One trial was three‐arm^23^ with two separate exercise intervention groups (moderate and high intensity). All included RCTs provided data on HRQoL that could be included in meta‐analysis.

### Study and population characteristics

The 12 included trials recruited a total of 1005 HFpEF patients. The study and population characteristics for each included trial are summarized in *Table*s 1 and 2. Seven trials were conducted in the United States, three in Europe, one in Australia and one in China. Patients' characteristics ranged widely across trials. Participant's median age was 68.5 years with a predominance of females (63%). Four trials defined HFpEF as an LVEF ≥ 45% and eight trials as ≥50%. The median sample size was 61 patients, two trials,^23^, ^24^ having more than 100 participants. Two trials^23^, ^25^ had a follow‐up of ≥6 months, the median follow‐up being 16 weeks across all trials. Nine trials undertook centre‐based CR delivery, and the remainder were home‐based. The dose of exercise intervention prescription varied considerably across studies with a range of programme duration of 12–52 weeks with two to four sessions per week of 20–60 min/session.

**Table 1:** Population characteristics of included trials.

First author, publication year	Sample size	LVEF (%), NYHA class	Age (years)		Sex (male)		BMI (kg/m^2^)		Hypertension		Diabetes mellitus	
			Mean ± *SD*		*n* (%)		Mean ± *SD*		*n* (%)		*n* (%)	
			ExCR	Control	ExCR	Control	ExCR	Control	ExCR	Control	ExCR	Control
Gary et al., 2004	28	≥45, II III	67 ± 11	69 ± 11	0 (0)	0 (0)	35 ± 6	32 ± 7	14 (88)	14 (88)	3 (19)	7 (44)
Andryukhin et al., 2010	75	≥5, I II III	66.5 (59 to 70)^a^	68 (57 to 72)^a^	12 (27)	14 (34)	30 (28.8 to 32)^a^	30 (28.4 to 31.3)^a^	NR (NR)	NR (NR)	NR (NR)	NR (NR)
Kitzman et al., 2010	46	≥50, II III	70 ± 6	69 ± 5	6 (17)	7 (9)	31.0 ± 6	31.0 ± 7	20 (87)	16 (84)	2 (8)	7 (32)
Smart et al., 2012	25	≥45, I II	67 ± 5.8	61.9 ± 6.9	7 (58)	6 (46)	31.1 ± 5.5	33.1 ± 7.3	2 (17)	2 (15)	2 (17)	2 (15)
Nolte et al., 2013	64	≥50, II III	64 ± 8.0	65 ± 6	20 (45)	8 (40)	31 ± 6	31 ± 4	38 (86)	17 (85)	7 (16)	2 (10)
Kitzman et al., 2013	47	≥50, II III	70 ± 7	70 ± 7	9 (28)	6 (20)	32.2 ± 6.7	32.0 ± 6.6	30 (94)	26 (84)	9 (28)	6 (19)
Fu T‐C et al., 2016	59	≥50, II III	60.5 ± 2.7	63.1 ± 2.6	20 (67)	18 (60)	NR	NR	25 (83)	13 (43)	19 (63)	6 (20)
Kitzman et al., 2016	92	≥50, II III	66.9 ± 5.5	66 ± 4.8	10 (20)	9 (18)	40.3 ± 7.1	38.4 ± 4.8	3 (6)	2 (4)	30 (59)	35 (71)
Lang et al., 2017	45	≥45, I II III	71.8 ± 9.9	76 ± 6.6	9 (36)	14 (56)	32.1 ± 6.3	32.2 ± 5.3	18 (72)	14 (56)	9 (36)	6 (24)
Brubaker et al., 2020	87	≥50, II III	70.3 ± 6.7	69.2 ± 6.2	14 (24)	8 (14)	30.1 ± 5.9	32.5 ± 6.6	NR (NR)	NR (NR)	NR (NR)	NR (NR)
Mentz et al., 2021	176	≥45, II III IV	72.7 ± 8.5	72.4 ± 7.8	39 (42)	34 (37)	35.4 ± 8.3	34.7 ± 9.3	89 (96)	86 (93)	62 (67)	41 (45)
Mueller et al., 2021	143	≥50, II III	70 ± 7.0	69 ± 10	40 (35)	19 (32)	30.0/31.1 ± 5.7/6.2	29.0 ± 4.7	99 (85)	51 (85)	32 (28)	14 (23)

Abbreviations: BMI, body mass index; ExCR, exercise‐based cardiac rehabilitation; LVEF, left ventricular ejection fraction; NR, not reported; NYHA, New York Heart Association; *SD*, standard deviation.

^a^ Median (interquartile range).

**Table 2:** Study characteristics of included trials.

Author (year)	Country	Follow‐up (weeks)	Delivery model	Exercise intervention	Exercise session duration	Exercise frequency	HRQoL measures	Results summary
Gary et al., 2004	US	12	Home‐based	Walking programme: 5 min warm‐up, walking, 5 min cool down.	Tailored for each individual	3 days/week	MLHFQ	MLHFQ overall score decreased with ExCR compared with control (*P* ≤ 0.05).
Andryukhin et al., 2010	Russia	24	Centre‐based	I Phase: Respiratory exercises. II Phase: Exercises for small muscle groups in sitting or standing positions, including weight training. III Phase: Exercises for large muscle groups in sitting or standing positions. IV Phase: Low‐intensity walking. V Phase: Moderate‐intensity walking.	Initial phase: 30 min/session. I to V Phase: duration personalised	Initial Phase: 4 days/week. I to V Phase: frequency personalized	MLHFQ	MLHFQ Overall scale decreased from (effect sizes (95% CI)) 54.5 (44–59) to 44.5 (35–47) & control group increased from 58 (49–65) to 61 (55–70).
Kitzman et al., 2010	US	16	Centre‐based	Aerobic endurance training: warm‐up, walking and cycling, cool down	60 min/session	3 days/week	MLHFQ, SF‐36	MLHFQ Physical health scale decreased (*P* ≤ 0.05).
Smart et al., 2012	Australia	16	Centre‐based	Aerobic endurance training (cycling)	30 min/session	3 days/week	MLHFQ	MLHFQ Overall scale showed no significant differences (*P* > 0.05).
Nolte et al., 2013	Germany	12	Centre‐based	Combined aerobic endurance (cycling) and resistance training (Bench press, leg press, leg curl, rowing machine, etc.).	Aerobic endurance training: 20–40 min/session. Resistance training: 15 repetitions/session.	Weeks 1–4: Aerobic endurance training 2 days/week; Weeks 5–12: Aerobic endurance training 3 days/week. Resistance training 2 days/week.	MLHFQ, SF‐36	MLHFQ overall scale and physical health scale decreased (*P* ≤ 0.05). SF‐36 physical functioning, bodily pain, general health perception, general mental health, vitality, social functioning, and physical and mental component scores increased (*P* ≤ 0.05).
Kitzman et al., 2013	US	16	Centre‐based	Aerobic endurance training: 10 min warm‐up, walking and cycling, 10 min cool‐down	60 min/session	3 days/week	MLHFQ, SF‐36	SF‐36 Physical and Emotional component scale increased (*P* ≤ 0.05). Scores of MLHFQ showed no significant difference (*P* > 0.05).
Kitzman et al., 2016	US	20	Centre‐based	Walking programme primarily	60 min/session	3 days/week	MLHFQ, KCCQ, SF‐36	Scores of MLHFQ, KCCQ, and SF‐36 showed no significant differences (*P* > 0.05).
Fu T‐C et al., 2016	China	12	Centre‐based	Aerobic endurance training: 3 min warm‐up, cycling, 3 min cool down	30 min/session	3 days/week	MLHFQ, SF‐36	MLHFQ overall scale decreased (*P* ≤ 0.05) SF‐36 scores (*P* ≤ 0.05).
Lang et al., 2017	UK	12	Home‐based	Walking, chair‐based exercise training, or the combination of walking and chair‐based exercise	NR	NR	MLHFQ, EQ‐5D	MLHFQ overall Scale decreased (−11.5, 95% CI ‐22.8 to 0.3) EQ‐5D index score increased (0.11, 95% CI ‐0.04 to 0.26)
Brubaker et al., 2020	US	16	Centre‐based	Endurance exercise training: 5 min warm‐up, walking and cycling, 10 min cool‐down	60 min/session	3 days/week	MLHFQ, SF‐36	MLHFQ overall scale showed no significant differences (*P* > 0.05) SF‐36 Physical function Scale increased (*P* ≤ 0.05).
Mentz et al., 2021	US	12	Centre‐based or home‐based	Outpatient sessions or low intensity walking and strengthening exercises at home on nonprogramme days	60 min/session at healthcare institution or top to 30 min/session at home	3 days/week	KCCQ, EQ‐5D	KCCQ overall scale and EQ‐5D visual analogue scale increased significantly (*P* ≤ 0.05).
Mueller et al., 2021	US	52	Hybrid	High‐intensity training: Bicycle aerobic endurance training; 10 min warm‐up, 4 × 4 min intervals, interspaced by 3 min cool down Moderate training: Bicycle aerobic endurance training	HIIT group: 38 min/session; MCT group: 40 min/session	HIIT group: 3 days/week; MCT group: 5 days/week	KCCQ	KCCQ overall scale increased significantly (95% CI: 2 to 19) in the moderate continuous training group in 12 months.

Abbreviations: HIIT, high‐intensity interval training; HRQoL, health‐related quality of life; KCCQ, Kansas City Cardiomyopathy Questionnaire; MCT, moderate continuous training; MLWHF, Minnesota Living with Heart Failure questionnaire; NR, not reported; SF‐36, Short Form‐36; UK, United Kingdom; US, United States.

A range of HRQoL outcomes were reported including the generic measures of the Short Form‐36 (SF‐36) (five trials) and the EuroQoL (EQ‐5D) (two trials) and the disease‐specific measures of the Minnesota Living with Heart Failure Questionnaire (MLHFQ) (nine trials) and the Kansas City Cardiomyopathy Questionnaire (KCCQ) (three3 trials).

### Risk of bias assessment

**Figure 2:**
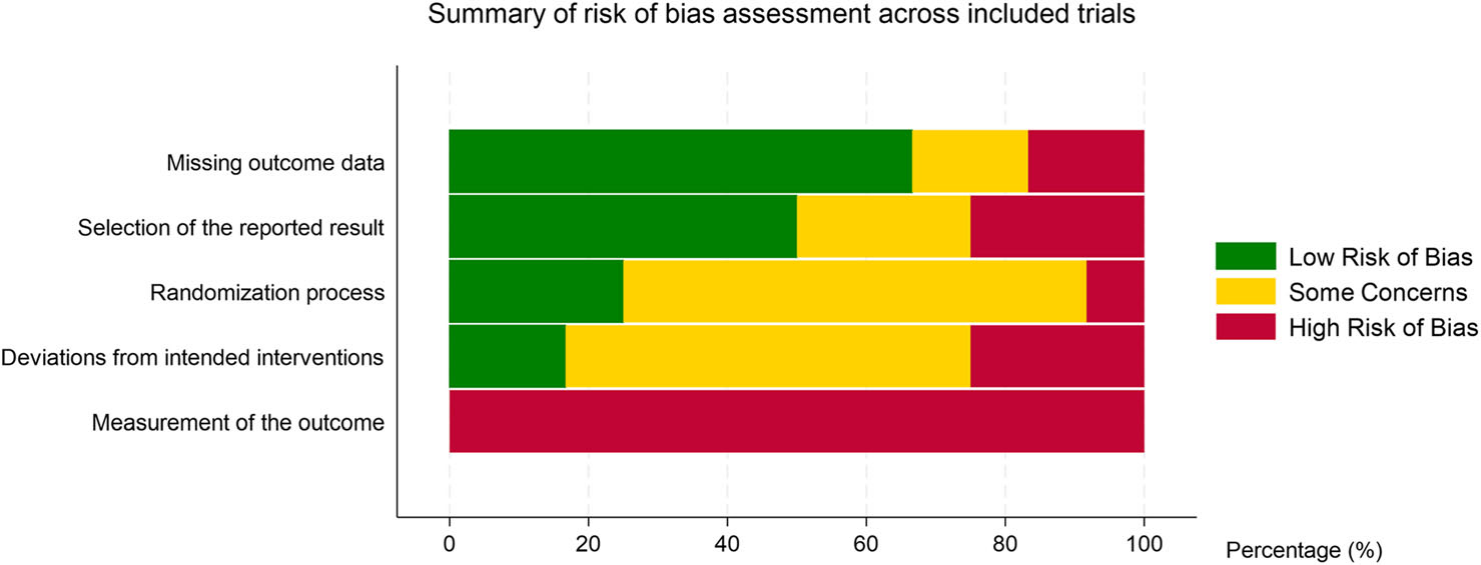
Summary of overall risk of bias across included studies.

Lack of reporting meant that risk of bias was judged to be uncertain across the majority of trials for the domains of randomization process (sequence generation and allocation concealment), deviations from intended intervention (failure to blind participants or carers and inappropriate statistical analysis used to estimate the effects) and selection of reported result (no protocol available, lack of complete outcome reporting and details of analysis method). Trials were judged to have a low risk for the ‘missing outcome data’ (8/12 trials) domain. All trials had high risk of ‘measurement of the outcome’ (see *Figure* 2 and Appendix S1).

### HRQoL findings

**Figure 3:**
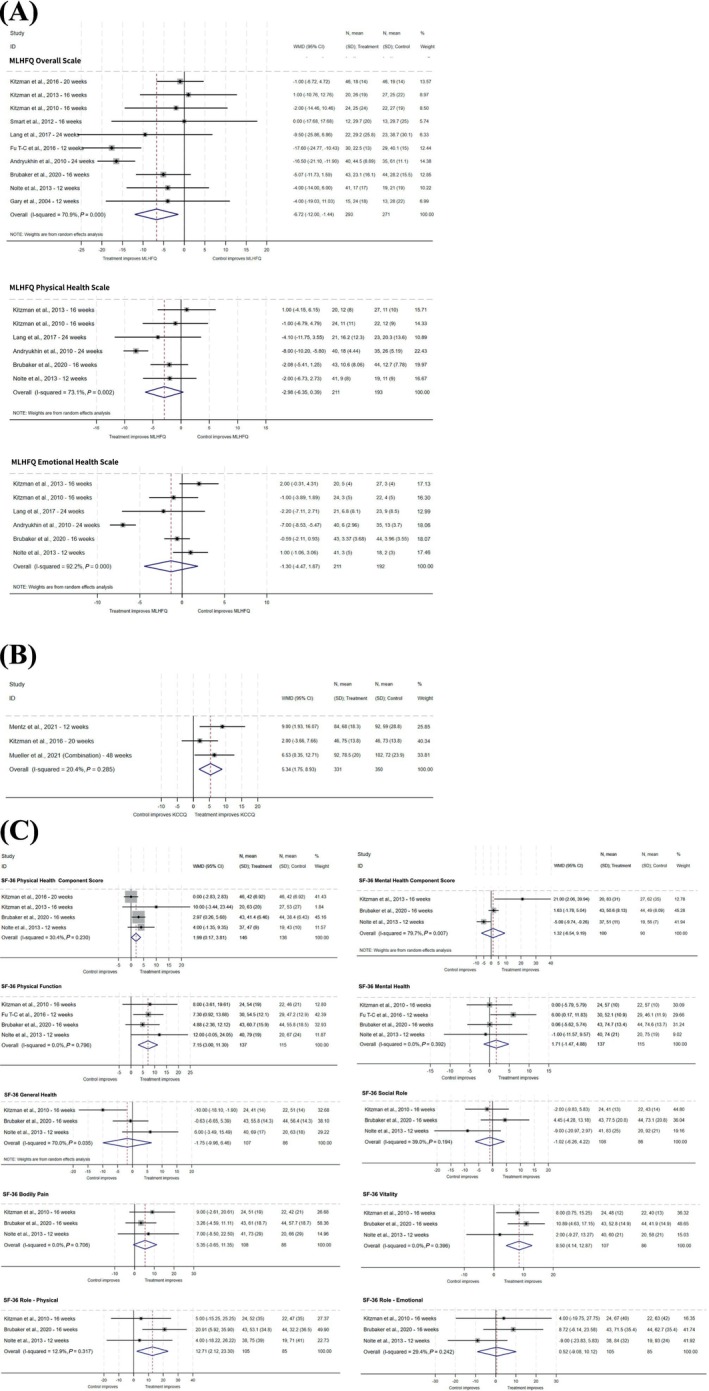
Forest plots of ExCR versus control for HRQoL in HFpEF patients (A) MLHFQ, (B) KCCQ and (C) SF‐36. 95% CI, 95% confidence interval; WMD, weighted mean difference.

*Table* 3 and *Figure* 3 provide a summary of pooled HRQoL results across trials.

**Table 3:** Summary of HRQoL meta‐analyses: ExCR vs. control.

HRQoL outcome	*N* studies (*N* patients)	Pooled result ExCR vs. control at follow‐up WMD (95% CI)^a^, *P* value	Statistical heterogeneity *I* ^2^ statistic	Outcome measure MCID
MLHFQ total	9 (495)	−6.72 (−12.00 to −1.44), 0.013	70.9% (high)	5.0 points
MLHFQ physical subscale	6 (360)	−2.98 (−6.35 to 0.39), 0.084	73.1% (high)	
MLHFQ mental subscale	6 (359)	−1.30 (−4.47 to 1.87), 0.421	92.2% (high)	
KCCQ overall summary score	3 (411)	5.44 (1.40 to 9.49), 0.008	20.4% (low)	5.0 points
SF‐36 physical component score	4 (282)	2.20 (−0.24 to 4.63), 0.077	30.4% (moderate)	1.0 point
SF‐36 mental health component score	3 (190)	1.32 (−6.54 to 9.19), 0.741	79.7% (high)	1.0 point

^a^ Random effect meta‐analysis model.

Abbreviations: EQ‐5D, EuroQoL; ExCR, exercise‐based cardiac rehabilitation; HRQoL, health‐related quality of life; KCCQ, Kansas City Cardiomyopathy Questionnaire; MCID, minimum clinically important difference; MLHFQ, Minnesota Living with Heart Failure questionnaire; *N*, number; SF‐36, Short‐Form‐36; WMD, weighted mean difference.

#### MLHFQ

Compared with no exercise control, at follow‐up, ExCR improved pooled WMD total MLHFQ score by −6.72 points (95% CI: −12.00 to −1.44) (see *Figure* 3A). There was weak evidence of an improvement in MLHFQ physical and emotional subscales. Statistical heterogeneity was high.

#### KCCQ

There was an improvement in KCCQ with ExCR (WMD: 5.34; 95% CI: 1.75 to 8.93) (see *Figure* 3B). Statistical heterogeneity was low.

#### SF‐36

Improvements were seen in SF‐36 physical component score (PCS) (WMD: 1.99; 95% CI: 0.17 to 3.81), in mental component score (MCS) (1.32, 95% CI: −6.54 to 9.19) and with ‘vitality scale’ (WMD: 8.5; 4.14 to 12.87) (see *Figure* 3C). Statistical heterogeneity ranged from low to high.

#### EQ‐5D

Pooled analysis was not possible as EQ‐5D data were reported using different metrics. Both Mentz et al.^24^ and Lang et al.^26^ had weak evidence of an improvement in HRQoL EQ‐5D visual analogue scale–ExCR versus control mean difference: −4 (95% CI: −9 to 0) and EQ‐5D index score–ExCR versus control mean difference: 0.11 (95% CI: −0.04 to 0.26), respectively.

### Exploration of statistical heterogeneity

There were sufficient trials to formally explore statistical heterogeneity for MLHFQ total score. For this outcome, subgroup analysis showed that mean exercise training session duration and trial sample size as potential predictors of the impact of ExCR (see Appendix S1). Trials of ≥60 min/session had evidence of a smaller ExCR effect (*P* < 0.01) than trials of duration of ≤60 min/session (WMD: −2.26; 95% CI: −6.12 to 1.61 vs. WMD: −16.05; 95% CI: −19.83 to −12.27, between group heterogeneity *P* < 0.001). Larger sample size group was associated with larger effects (WMD: −10.58; 95% CI: −13.46 to −7.89, *P* = 0.02). No impact was seen for trial publication date (<10 years old: WMD: −6.85, 95% CI: −10.47 to −3.23 vs. >10 years old: WMD: −10.74, 95% CI: −14.30 to −7.17, between group heterogeneity *P* = 0.134), HFpEF definition (LVEF ≥ 50%: WMD: −9.15; 95% CI: −11.79 to −6.51 vs. LVEF ≥ 45%: WMD: −4.68, 95% CI: −14.07 to 4.70, between group heterogeneity *P* = 0.369) or ExCR mode of delivery (centre‐based–WMD: −8.95, 95% CI: −11.56 to −6.34; home‐based–WMD: −6.52, 95% CI: −17.59 to 4.55, between group heterogeneity *P* = 0.675).

Sensitivity analyses indicated that bias domains of deviation from intended interventions, missing outcome data and selection of the reported results may all be sources of high statistical heterogeneity. Except for the domain of missing outcome data, the ‘low‐risk’ group was associated with low statistical heterogeneity. The heterogeneity tests between all groups were statistically significant (*P* ≤ 0.05) (see Appendix S1).

### Publication bias

There was no evidence of funnel asymmetry for total MLHFQ score (Egger test: *P* = 0.193, see Appendix S1).

## Discussion

This systematic review and meta‐analyses of randomized trials presents evidence of ExCR in improving HRQoL of people with HFpEF compared with no exercise control. Our study showed HRQoL improvements are consistent across both disease‐specific (i.e., MLHFQ and KCCQ) and generic (i.e., SF‐36 and EQ‐5D) outcomes and appear to be driven by improvements in both physical and mental well‐being. Importantly, the magnitude of improvement was not only statistically significant but also clinically meaningful. The mean improvements in both MLHFQ and KCCQ exceeded the reported minimally important clinical difference for both instruments of 5 points (see *Table* 2).^27^, ^28^

Our finding of the beneficial effects of ExCR on HRQoL is consistent with a previous meta‐analysis.^11^ However, in contrast to the present study, this previous analysis was limited to a single HRQoL outcome (e.g., MLHFQ) and included a smaller number of RCTs.

Differences in patient population (e.g., HFpEF definition and sex distributions), nature of ExCR interventions (e.g., duration, intensity) across included trials and risk of bias, are likely to have contributed to high levels of statistical heterogeneity seen in this review. We undertook extensive subgroup and sensitivity analyses to explore this heterogeneity. While analyses showed that the benefits of ExCR appeared to be consistent across trial definition of HFpEF (LVEF ≥45% vs. ≥50%), the effect size of trials varied by trial mean exercise session duration and risk of bias. Trials with shorter exercise session duration (<60 min/session) were associated with larger HRQoL gains than those with longer duration. This finding may reflect shorter training sessions to avoid excessive fatigue, allowing patients to benefit more fully from ExCR and accumulate more health benefits.^29^ Furthermore, psychologically shorter training sessions may facilitate better adherence. Physical fatigue, mental stress and the monotony of repetitive exercises were significant psychological factors that can affect patients undergoing ExCR.^30^ This finding emphasises the importance of tailoring ExCR programmes to the specific needs of patients.

## Strengths and limitations

Our study provides a comprehensive analysis of the impact of ExCR in people with HFpEF. However, we also recognize limitations in our study. First, included trials were small and demonstrated substantial clinical heterogeneity in terms of (1) their population definition (e.g., definition of HFpEF of ≥45% vs ≥50%, level and nature of comorbidities); (2) nature of the ExCR intervention (e.g., exercise only vs. comprehensive CR programmes, different modes and intensity of exercise, different delivery settings—centre vs. home); and (3) HRQoL assessment (e.g., disease specific vs. generic measure). We undertook random effects meta‐analysis and pre‐specified subgroup and sensitivity analyses to examine the impact of this heterogeneity. Second, all included trials were open label so that patients knew whether they were allocated to intervention or control. As a patient‐reported outcome, HRQoL is subject to reporting bias and placebo effects.^31^ Third, some HRQoL outcomes were not consistently collected or reported across trials. For example, EQ‐5D was only assessed in two included trials, each reporting different outcome metrics resulting in data pooling not being possible. Four, lack of detailed methodological reporting by trials made the risk of bias assessment difficult. Finally, the high levels of statistical heterogeneity (i.e., *I* ^2^ > 75%) observed across HRQoL outcomes may have made our findings unreliable. However, our subgroup and sensitivity analyses did show that this heterogeneity was at least partly explainable and driven by trial risk of bias and variation in exercise session duration.

## Implications for future practice, policy and research

As recently highlighted by the intriguingly entitled editorial: *‘I don't wanna live forever’—importance of quality of life in heart failure patients*, HRQoL is now a recognized key target for HF management alongside the traditional focus of drugs and medical devices on reducing hospitalization and mortality.^32^ The European Association of Preventive Cardiology recently published their consensus paper on HRQoL^33^ and in 2020 the US Food and Drug Administration accepted KCCQ as an outcome that can be used in drug approval for HF.^16^

Our pooled analyses of RCTs are supportive of the importance of ExCR referral and participation for people with HFpEF to provide clinically relevant improvements in their HRQoL. However, our analyses also highlight the need for well reported fully powered RCTs of ExCR with adequate follow‐up (≥6 months). Future trials should consistently collect and report both disease‐specific and generic HRQoL measures.

## Conclusions

This systematic review and meta‐analysis identified randomized trials assessing ExCR compared with no exercise control for people with HFpEF. Although there was evidence of improvement in both generic and disease HRQoL with ExCR participation, included trials were small and heterogenous with a wide range of study populations, exercise interventions and outcome measures. Four older trials defined HFpEF as a LVEF of ≥45% while eight more recent trials use the ESC 2023 definition of ≥50%. Well reported, fully powered multicentre RCTs of ExCR with longer follow‐up are needed to confirm the HRQoL benefits of ExCR in HFpEF.

## Conflict of interest statement

C. D. and Y. G. report no conflict of interest. R. S. T. is currently co‐chief investigator for the NIHR HTA funded REACH‐HFpEF trial (grant reference NIHR130487) and a co‐author/investigator on the Lang et al. (2017) randomized trial included in this review.

## Funding

No specific funding was received for this study. CD undertook this study as completion of the University of Glasgow, Masters of Public Health degree.

## Supporting information


**Appendix S1.** Supporting Information.

## References

[1] Groenewegen A , Rutten FH , Mosterd A , Hoes AW . Epidemiology of heart failure. Eur J Heart Fail 2020;22:1342‐1356. doi:10.1002/ejhf.1858 32483830 PMC7540043

[2] McDonagh TA , Metra M , Adamo M , Gardner RS , Baumbach A , Böhm M , *et al*. 2023 Focused update of the 2021 ESC guidelines for the diagnosis and treatment of acute and chronic heart failure. Eur Heart J 2023;44:3627‐3639. doi:10.1093/eurheartj/ehad195 37622666

[3] Dunlay SM , Roger VL , Redfield MM . Epidemiology of heart failure with preserved ejection fraction. Nat Rev Cardiol 2017;14:591‐602. doi:10.1038/nrcardio.2017.65 28492288

[4] Nichols GA , Qiao Q , Deruaz‐Luyet A , Kraus BJ . Hospitalization and mortality in heart failure with preserved ejection fraction: real‐world data from a US integrated healthcare delivery system. Eur Heart J 2021;42:ehab724.0729. doi:10.1093/eurheartj/ehab724.0729

[5] Ventoulis I , Kamperidis V , Abraham MR , Abraham T , Boultadakis A , Tsioukras E , *et al*. Differences in health‐related quality of life among patients with heart failure. Medicina (Kaunas) 2024;60:109. doi:10.3390/medicina60010109 38256370 PMC10818915

[6] Wintrich J , Kindermann I , Ukena C , Selejan S , Werner C , Maack C , *et al*. Therapeutic approaches in heart failure with preserved ejection fraction: past, present, and future. Clin Res Cardiol 2020;109:1079‐1098. doi:10.1007/s00392-020-01633-w 32236720 PMC7449942

[7] Redfield MM , Borlaug BA . Heart failure with preserved ejection fraction: a review. JAMA 2023;329:827‐838. doi:10.1001/jama.2023.2020 36917048

[8] Patti A , Merlo L , Ambrosetti M , Sarto P . Exercise‐based cardiac rehabilitation programs in heart failure patients. Heart Fail Clin 2021;17:263‐271. doi:10.1016/j.hfc.2021.01.007 33673950

[9] Molloy CD , Long L , Mordi IR , Bridges C , Sagar VA , Davies EJ , *et al*. Exercise‐based cardiac rehabilitation for adults with heart failure—2023 Cochrane systematic review and meta‐analysis. Eur J Heart Fail 2023;25:2263‐2273. doi:10.1002/ejhf.3046 37850321

[10] Taylor RS , Walker S , Smart NA , Piepoli MF , Warren FC , Ciani O , *et al*. Impact of exercise rehabilitation on exercise capacity and quality‐of‐life in heart failure: individual participant meta‐analysis. J Am Coll Cardiol 2019;73:1430‐1443. doi:10.1016/j.jacc.2018.12.072 30922474 PMC8351793

[11] Fukuta H , Goto T , Wakami K , Kamiya T , Ohte N . Effects of exercise training on cardiac function, exercise capacity, and quality of life in heart failure with preserved ejection fraction: a meta‐analysis of randomized controlled trials. Heart Fail Rev 2019;24:535‐547. doi:10.1007/s10741-019-09774-5 31032533

[12] Page MJ , McKenzie JE , Bossuyt PM , Boutron I , Hoffmann TC , Mulrow CD , *et al*. The PRISMA 2020 statement: an updated guideline for reporting systematic reviews. BMJ 2021;372: doi:10.1136/bmj.n71 PMC8005924 33782057

[13] Ware JE , Sherbourne CD . The MOS 36‐item short‐form health survey (SF‐36): i. Conceptual framework and item selection. Med Care 1992;30:473‐483. doi:10.1097/00005650-199206000-00002 1593914

[14] EuroQoL Group . EuroQoL: a new facility for the measurement of health‐related quality of life. Health Policy 1990;16:199‐208. doi:10.1016/0168-8510(90)90421-9 10109801

[15] Heo S , Moser DK , Riegel B , Hall LA , Christman N . Testing the psychometric properties of the Minnesota Living with Heart Failure questionnaire. Nurs Res 2005;54:265‐272. doi:10.1097/00006199-200507000-00009 16027569

[16] US Food and Drug Administration . DDT COA #000084: Kansas City Cardiomyopathy Questionnaire (KCCQ). US Food and Drug Administration; 2020. Available from: https://www.fda.gov/drugs/clinical‐outcome‐assessment‐coa‐qualificationprogram/ddt‐coa‐000084‐kansas‐city‐cardiomyopathy‐questionnaire‐kccq. Accessed 18 August 2025

[17] Higgins JPT , Savović J , Page MJ , Sterne JAC . Revised Cochrane risk‐of‐bias tool for randomized trials (RoB 2). Cochrane Bias Methods; 2019. Available from: https://sites.google.com/site/riskofbiastool/welcome/rob‐2‐0‐tool/current‐version‐of‐rob‐2, Accessed 18 August 2025. doi:10.1016/j.ajogmf.2019.100048

[18] Lin L , Chu H . Quantifying publication bias in meta‐analysis. Biometrics 2018;74:785‐794. doi:10.1111/biom.12817 29141096 PMC5953768

[19] Rücker G , Cates CJ , Schwarzer G . Methods for including information from multi‐arm trials in pairwise meta‐analysis. Res Synth Methods 2017;8:392‐403. doi:10.1002/jrsm.1259 28759708

[20] Higgins JPT , Thomas J , Chandler J , Cumpston M , Li T , Page MJ , et al. Cochrane handbook for systematic reviews of interventions, version 6.4. 2023. Available from: https://training.cochrane.org/handbook/current/chapter‐10. Accessed 18 August 2025

[21] Palau P , Domínguez E , Núñez E , Schmid JP , Vergara P , Ramón JM , *et al*. Effects of inspiratory muscle training in patients with heart failure with preserved ejection fraction. Eur J Prev Cardiol 2014;21:1465‐1473. doi:10.1177/2047487313498832 23864363

[22] Palau P , Domínguez E , López L , Ramón JM , Heredia R , González J , *et al*. Inspiratory muscle training and functional electrical stimulation for treatment of heart failure with preserved ejection fraction: the TRAINING‐HF trial. Rev Esp Cardiol (Engl Ed) 2019;72:288‐297. doi:10.1016/j.rec.2018.01.010 29551699

[23] Mueller S , Winzer EB , Duvinage A , Gevaert AB , Edelmann F , Haller B , *et al*. Effect of high‐intensity interval training, moderate continuous training, or guideline‐based physical activity advice on peak oxygen consumption in patients with heart failure with preserved ejection fraction: a randomized clinical trial. JAMA 2021;325:542‐551. doi:10.1001/jama.2020.26812 33560320 PMC7873782

[24] Mentz RJ , Whellan DJ , Reeves GR , Pastva AM , Duncan P , Upadhya B , *et al*. Rehabilitation intervention in older patients with acute heart failure with preserved versus reduced ejection fraction. JACC: Heart Fail 2021;9:747‐757. doi:10.1016/j.jchf.2021.05.007 34246602 PMC8487922

[25] Andryukhin A , Frolova E , Vaes B , Degryse J . The impact of a nurse‐led care programme on events and physical and psychosocial parameters in patients with heart failure with preserved ejection fraction: a randomized clinical trial in primary care in Russia. Eur J Gen Pract 2010;16:205‐214. doi:10.3109/13814788.2010.527938 21073267

[26] Lang CC , Smith K , Wingham J , Eyre V , Greaves CJ , Warren FC , *et al*. A randomised controlled trial of a facilitated home‐based rehabilitation intervention in patients with heart failure with preserved ejection fraction and their caregivers: the REACH‐HFpEF pilot study. BMJ Open 2018;8:e019649. doi:10.1136/bmjopen-2017-019649 PMC5893929 29632081

[27] Spertus JA , Jones PG , Sandhu AT , Arnold SV . Interpreting the Kansas City cardiomyopathy questionnaire in clinical trials and clinical care: JACC state‐of‐the‐art review. J Am Coll Cardiol 2020;76:2379‐2390. doi:10.1016/j.jacc.2020.09.542 33183512

[28] American Thoracic Society . Minnesota Living with Heart Failure Questionnaire. 2004. Available from: https://qol.thoracic.org/sections/instruments/ko/pages/mlwhfq.htm. Accessed 18 August 2025

[29] Fry AC , Kraemer WJ , van Borselen F , Lynch JM , Marsit JL , Roy EP , *et al*. Performance decrements with high‐intensity resistance exercise overtraining. Med Sci Sports Exerc 1994;26:1165‐1173. doi:10.1249/00005768-199409000-00015 7808252

[30] Denollet J . Emotional distress and fatigue in coronary heart disease: the Global Mood Scale (GMS). Psychol Med 1993;23:111‐121. doi:10.1017/s0033291700038903 8475198

[31] Tu Y , Zhang L , Kong J . Placebo and nocebo effects: from observation to harnessing and clinical application. Transl Psychiatry 2022;12:524. doi:10.1038/s41398-022-02293-2 36564374 PMC9789123

[32] Schmid JP . I don't wanna live forever—importance of quality of life in heart failure patients. Eur J Prev Cardiol 2024;31:1425‐1426. doi:10.1093/eurjpc/zwae112 38487892

[33] Volterrani M , Halasz G , Adamopoulos S , Agostoni PG , Butler J , Coats AJS , *et al*. Quality of life in heart failure: the heart of the matter—a scientific statement of the Heart Failure Association and the European Association of Preventive Cardiology. Eur J Prev Cardiol In press; doi:10.1093/eurjpc/zwad400 40070307

