# Supplementary material for: Cardiac rehabilitation and health‐related quality of life in preserved ejection fraction heart failure: A meta‐analysis
Source: ESC Heart Fail. 2025 Sep 3;12(6):3929–39. doi: 10.1002/ehf2.15404 (PMC12719872; doi:10.1002/ehf2.15404)
Supplement: Supplementary file 1 — Appendix S1. Supporting Information. [file EHF2-12-3929-s001.pdf]

SUPPLEMENTARY APPENDIX

Search strategy

1. ‘heart failure with preserved ejection fraction’:ti,ab OR ‘HFpEF’:ti,ab OR ‘diastolic heart failure’:ti,ab OR ‘heart failure’:ti,ab OR ‘HF’:ti,ab
2. ‘exercise’:ti,ab OR ‘exercise therapy’:ti,ab OR ‘exercise based cardiac rehabilitation’:ti,ab OR ‘rehabilitation’:ti,ab
3. ‘quality of life’:ti,ab OR ‘health related quality of life’:ti,ab OR ‘qol’:ti,ab OR ‘HRQOL’:ti,ab OR ‘HRQL’:ti,ab OR ‘patient reported outcome*’: ti,ab OR ‘PRO’: ti,ab OR ‘self-reported outcome*’ : ti,ab OR ‘EQ5D’:tw OR ‘EQ 5D’:tw OR ‘EuroQoL’:tw OR ‘Kansas City Cardiomyopathy Questionnaire’:tw OR ‘KCCQ’:tw OR ‘Minnesota Living with Heart Failure Questionnaire’:tw OR ‘MLWHFQ’:tw OR ‘SF-36’:tw OR ‘SF-12’:tw
4. ‘randomized controlled trial’:ti,ab OR ‘controlled clinical trial’:ti,ab OR ‘randomized’:ti,ab OR ‘placebo’:ti,ab OR ‘randomly’:ti,ab OR ‘trial’:ti,ab OR ‘groups’:ti,ab OR ‘RCTs’:ti,ab OR ‘RCT’:ti,ab
5. 1 AND 2 AND 3 AND 4
6. Limit 5 to (randomized controlled trial OR clinical trial)
7. Limit 6 to full text
8. Limit 7 to English and Chinese languages

Excluded trials and reasons

Trials without independent intervention group:

Hwang, R., Bruning, J., Morris, N. R., Mandrusiak, A., & Russell, T. (2017). Home-based telerehabilitation is not inferior to a centre-based program in patients with chronic heart failure: a randomised trial. Journal of Physiotherapy, 63:101–107.

Ma, C., Zhou, W., Jia, Y., & Tang, Q. (2022). Effects of home-based Baduanjin combined with elastic band exercise in patients with chronic heart failure. European Journal of Cardiovascular Nursing, 21: 587–596.

Hwang, R., Morris, N. R., Mandrusiak, A., Bruning, J., Peters, R., Korczyk, D., & Russell, T. (2019). Cost-Utility Analysis of Home-Based Telerehabilitation Compared With Centre-Based Rehabilitation in Patients With Heart Failure. Heart, Lung & Circulation, 28:1795–1803.

Gary, R. A., Dunbar, S. B., Higgins, M. K., Musselman, D. L., & Smith, A. L. (2010). Combined exercise and cognitive behavioral therapy improves outcomes in patients with heart failure. Journal of Psychosomatic Research, 69:119–131

Chan, C., Tang, D., & Jones, A. (2008). Clinical outcomes of a Cardiac Rehabilitation and Maintenance Program for Chinese patients with congestive heart failure. Disability and Rehabilitation, 30:1245–1253.

Trial with protocol only:

Koifman, E., Grossman, E., Elis, A., Dicker, D., Koifman, B., Mosseri, M., Kuperstein, R., Goldenberg, I., Kamerman, T., Levine-Tiefenbrun, N., & Klempfner, R. (2014). Multidisciplinary rehabilitation program in recently hospitalized patients with heart failure and preserved ejection fraction: rationale and design of a randomized controlled trial. American Heart Journal, 168: 830–7.e1.

No full text available:

Windy Alonso, Kevin Kupzyk, Joseph Norman, Scott Lundgren, Merry Lindsey, Alfred Fisher, Sara Bills, Steven Keteyian, Bunny Pozehl, Long-term Adherence To Exercise In Adults With Preserved Ejection Fraction Heart Failure（2022, Journal of Cardiac Failure, 28: S5-S6,

Interventions that did not meet inclusion criteria:

Palau, P., Domínguez, E., López, L., Heredia, R., González, J., Ramón, J. M., Serra, P., Santas, E., Bodi, V., Sanchis, J., Chorro, F. J., & Núñez, J. (2016). inspiratory muscle training and functional electrical stimulation for treatment of heart failure with preserved ejection fraction: Rationale and study design of a prospective randomized controlled trial. Clinical cardiology, 39(8), 433–439.

Palau, P., Domínguez, E., Núñez, E., Schmid, J.P., Vergara, P., Ramón, J.M., Mascarell, B., Sanchis, J., Chorro, F.J. and Núñez, J. (2014) 'Effects of inspiratory muscle training in patients with heart failure with preserved ejection fraction', European Journal of Preventive Cardiology, 21:1465-1473.

Publications for included trial that did not provide additional data:

Edelmann, F., Gelbrich, G., Düngen, H. D., Fröhling, S., Wachter, R., Stahrenberg, R., Binder, L., Töpper, A., Lashki, D. J., Schwarz, S., Herrmann-Lingen, C., Löffler, M., Hasenfuss, G., Halle, M., & Pieske, B. (2011). Exercise training improves exercise capacity and diastolic function in patients with heart failure with preserved ejection fraction: results of the Ex-DHF (Exercise training in Diastolic Heart Failure) pilot study. Journal of the American College of Cardiology, 58:1780–1791.

Table. Risk of bias assessment of included trials

Author (year)	Randomisation process	Deviations from intended interventions	Missing outcome data	Measurement of the outcome	Selection of the reported result
Gary et al., 2004	Some Concerns	Some Concerns	High Risk	High Risk	High Risk
Andryukhin et al., 2010	Low risk	Some Concerns	Some Concerns	High Risk	Some Concerns
Kitzman et al., 2010	Some Concerns	High Risk	Low Risk	High Risk	Low Risk
Smart et al., 2012	Some Concerns	Low Risk	Low Risk	High Risk	Low Risk
Nolte et al., 2013	Some Concerns	Some Concerns	Low Risk	High Risk	Low Risk
Kitzman et al., 2013	Some Concerns	Some Concerns	Some Concerns	High Risk	High Risk
Fu T-C et al., 2016	Some Concerns	Some Concerns	Low Risk	High Risk	Some Concerns
Kitzman et al., 2016	Some Concerns	Low Risk	Low Risk	High Risk	High Risk
Lang et al., 2017	High Risk	Some Concerns	High Risk	High Risk	Some Concerns
Brubaker et al., 2020	Some Concerns	High Risk	Low Risk	High Risk	Low Risk

Mentz et al., 2021	Low risk	High Risk	Low Risk	High Risk	Low Risk
Mueller et al., 2021	Low risk	Some Concerns	Low Risk	High Risk	Low Risk

RoB 2: Revised Cochrane risk-of-bias tool for randomized trials.

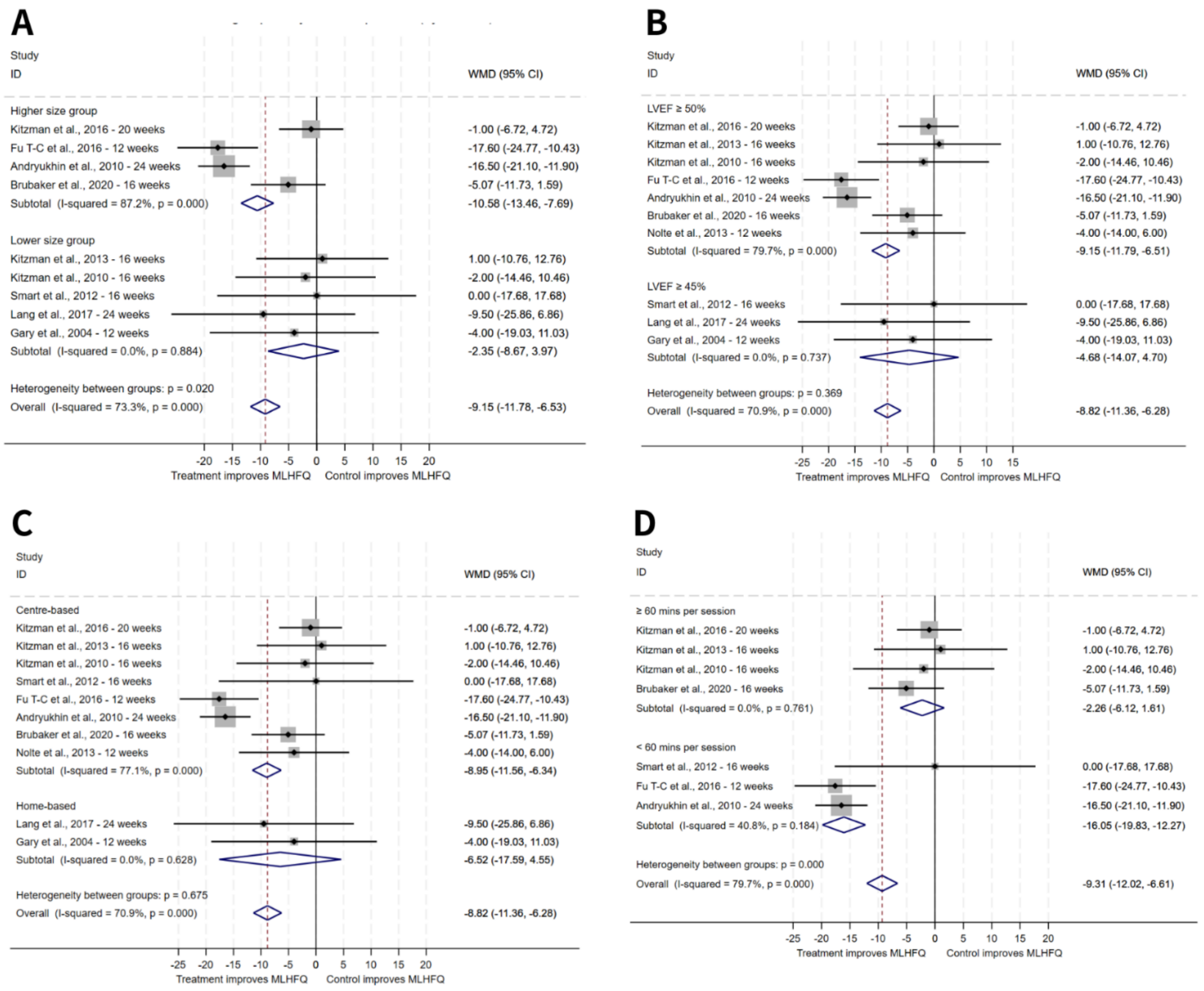

Figure. Forest plot of subgroup analysis of MLHFQ (A) sample size, (B) HFpEF diagnosis criteria, (C) CR delivery Model, (D) exercise training. SD, standard deviation; WMD, weighted mean difference; 95% Cl, 95% confidence interval.

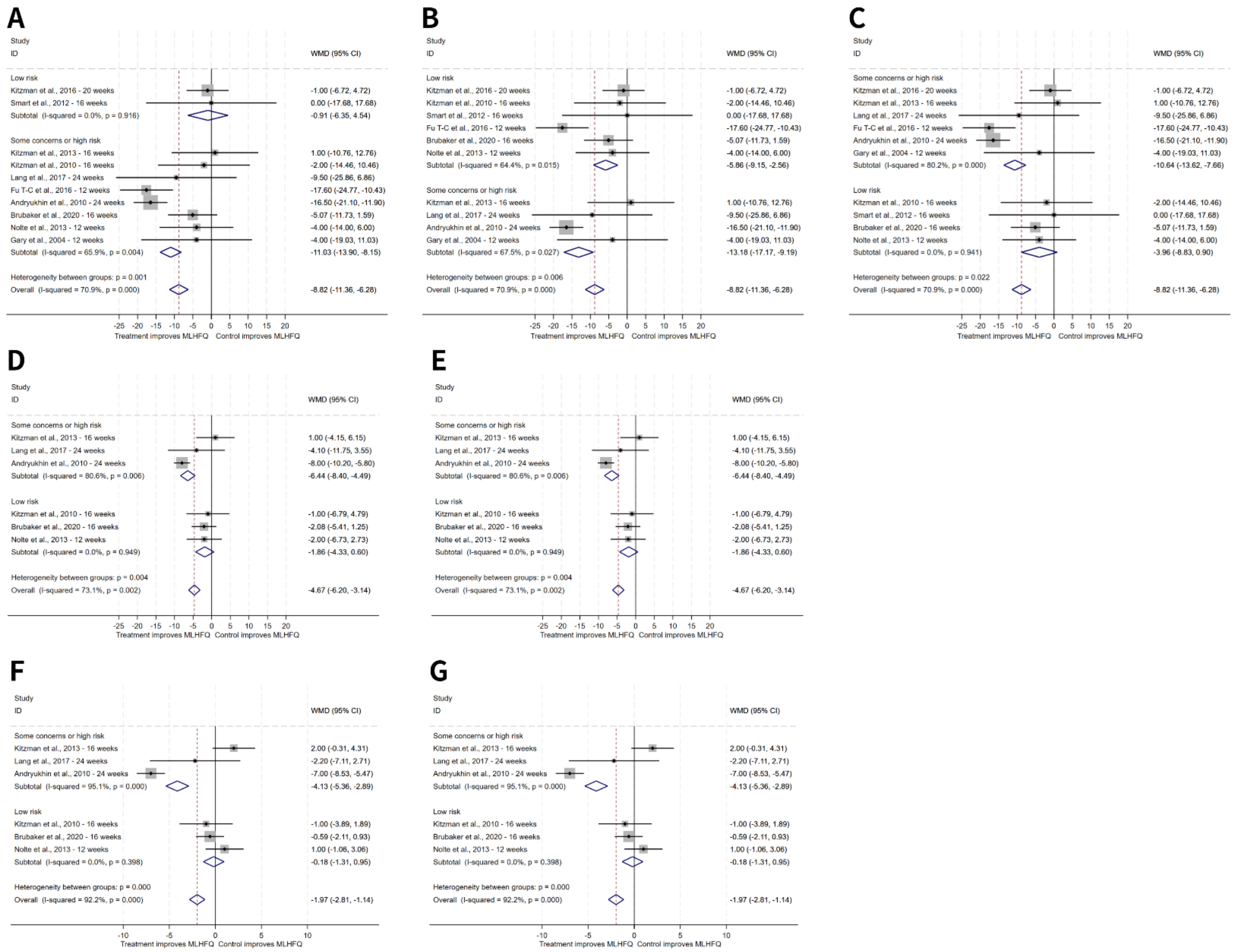

Figure. Sensitivity analysis of MLHFQ total score by risk of bias domain: (A) Deviations from intended interventions, (B) Missing outcome data, (C) Selection of reported results; MLHFQ Physical scale: (D) Missing outcome data, (E) Selection of Reported Results; and MLHFQ Emotional scale: (F) Missing Outcome Data, (G) Selection of reported Results. WMD, weighted mean difference; 95% Cl, 95% confidence interval.

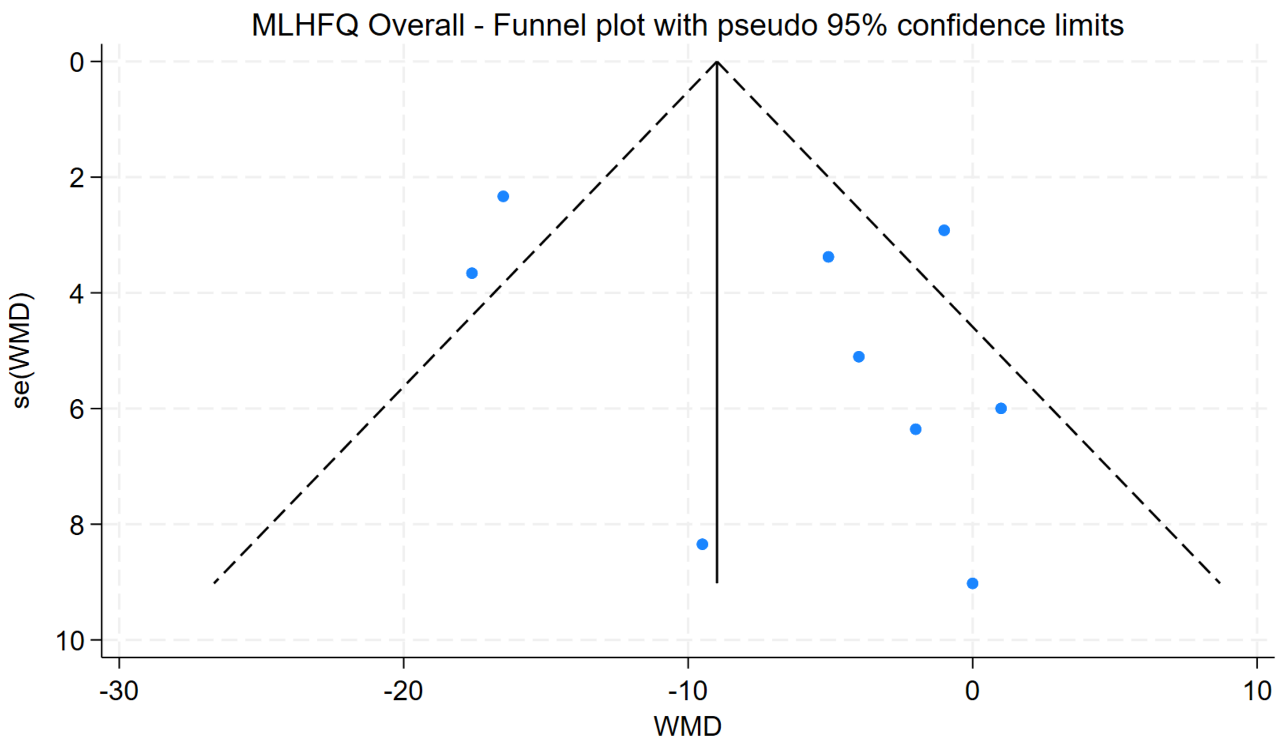

Figure. Meta funnel plot for MLHFQ total score

se: standard error; WMD: weighted mean difference.
